# A Rare Case of Perrault Syndrome with Auditory Neuropathy Spectrum Disorder: Cochlear Implantation Treatment and Literature Review

**DOI:** 10.3390/audiolres11040055

**Published:** 2021-11-13

**Authors:** Francesca Forli, Luca Bruschini, Beatrice Franciosi, Roberta Battini, Gemma Marinella, Stefano Berrettini, Francesco Lazzerini

**Affiliations:** 1 Otolaryngology, Audiology and Phoniatrics Unit, University of Pisa, 56100 Pisa, Italy; l.bruschini@gmail.com (L.B.); beatrice.franciosi.pr@gmail.com (B.F.); s.berrettini@med.unipi.it (S.B.); francilazzerini@gmail.com (F.L.); 2 Department of Surgical, Medical and Molecular Pathology and Critical Care Medicine, University of Pisa, 56100 Pisa, Italy; 3 Department of Developmental Neuroscience, IRCCS Fondazione Stella Maris, 56100 Pisa, Italy; rbattini@fsm.unipi.it (R.B.); gmarinella@fsm.unipi.it (G.M.); 4 Department of Clinical and Experimental Medicine, University of Pisa, 56100 Pisa, Italy; 5 Department of Clinical Science, Intervention and Technology, Karolinska Institutet, 17177 Stockholm, Sweden

**Keywords:** auditory neuropathy spectrum disorder, Perrault syndrome, cochlear implant

## Abstract

Perrault syndrome (PRLTS) is a rare autosomal recessive disorder characterised by ovarian failure in females and sensorineural hearing loss (SNHL) in both genders. In the present paper we describe a child affected by PRLTS3, due to CLPP homozygous mutations, presenting auditory neuropathy spectrum disorder (ANSD) with bilateral progressive SNHL. This is the first case reported in the literature of an ANSD in PRLTS3. CLPP is a nuclear encoded mitochondrial protease directed at the mitochondrial matrix. It is encoded on chromosome 19. This protease participates in mitochondrial protein quality control by degrading misfolded or damaged proteins, thus maintaining the normal metabolic function of the cell. In PRLTS3, the peptidase activity of CLPP is suppressed. Neurological impairments involved in PRLTS3 suggest that the pathogenic mutations in CLPP might trigger a mitochondrial dysfunction. A comprehensive description of the clinical and audiological presentation, as well as the issues related to cochlear implant (CI) procedure and the results, are addressed and discussed. A brief review of the literature on this topic is also provided.

## 1. Introduction

Perrault syndrome (PRLTS) is a rare autosomal recessive disorder characterised by ovarian failure in females and sensorineural hearing loss (SNHL) in both genders [1]. The initial expression of PRLTS is commonly represented by SNHL, and it can be followed by neurological abnormalities, typically motor and sensory neuropathy, muscle weakness and atrophy, cerebellar ataxia, limited eye movements, nystagmus, dyspraxia, as well as intellectual deficit, developmental delay and seizures. Magnetic resonance imaging (MRI) may be useful to detect cerebral leukodystrophy and cerebellar atrophy [2,3,4].

The genes involved in PRLTS expression are HSD17B4, HARS2, CLPP, LARS2, TWNK and ERAL1, defining, respectively, the subtypes PRLTS1 (OMIM# 233400), PRLTS2 (OMIM# 614926), PRLTS3, PRLTS4 (OMIM# 615300), PRLTS5 (OMIM# 616138) and PRLTS6 (OMIM# 617565) [5]. However, approximately 60% of patients with the clinical aspect of PRLTS lack a molecular diagnosis [6].

In PRLTS, SNHL is generally reported to be due to a cochlear dysfunction, and only three cases due to a neural deficit (auditory neuropathy) are reported in the scientific literature, two cases with PRTLS5, associated with TWNK mutation [7] and a further case in PRTLS2, associated with HARS2 mutation [8].

In the present paper we describe a child affected by PRLTS3, due to CLPP homozygous mutations, presenting auditory neuropathy spectrum disorder (ANSD) with bilateral progressive SNHL. This is the first case reported in the literature of an ANSD in PRLTS3. A comprehensive description of the clinical and audiological presentation, as well as the issues related to cochlear implant (CI) procedure and the results, are addressed and discussed. A brief review of the literature on this topic is also provided.

## 2. Case Report

The case presentation is compliant with the SCARE guidelines [9].

A female infant from consanguineous Senegalese parents was born preterm (36 weeks of gestational age) by caesarean section due to alteration of cardiotocography tracking. During the pregnancy a severe intrauterine growth restriction occurred from the second trimester. Body weight at birth was 1350 g, and APGAR score (an acronym for and index that evaluates the Appearance (skin colour), Pulse (heart rate), Grimace (reflex irritability), Activity (muscle tone), and Respiration of a newborn) was 2 in the first minute of life and which increased at 7 after 10 min. The child underwent cardiopulmonary resuscitation manoeuvres and tracheal intubation and was admitted to the neonatal intensive care unit for ventilation support. After 7 weeks, given the improvement of her general condition, the baby was discharged from the hospital. While hospitalized, the baby underwent newborn hearing screening, according to the protocol of the Tuscan region [10,11] and resulted pass bilaterally both at transient evoked otoacoustic emissions (TEOAE) and automatic auditory brainstem response (AABR) testing.

Given a retardation of her neuromotor and linguistic development, the baby was entrusted to the infantile neuropsychiatric service at 24 months of age. During this period of hospitalization, the infant underwent brain magnetic resonance, genetic testing and an audiologic revaluation. She presented global developmental delay involving the motor, cognitive and linguistic areas. However, the neurological examination did not reveal any specific neurological signs.

Brain magnetic resonance imaging (MRI) showed leukoencephalopathy and suggested a diagnostic hypothesis of mitochondrial disease.

Muscle biopsy and genetic tests showed signs suggestive of mitochondrial pathology due to a homozygous mutation of CLPP gene (mut. Homozygosity C.425C> T/p.Pro142Leu), defining a picture of PRLST3. Both the parents were heterozygous for the same mutation.

The audiologic assessment performed at 24 months revealed severe-to-profound hearing loss, with a free field threshold at behavioural audiometry around 95 dB (pure tone audiometry between 0.5 and 1-2-4KHz), with no sound detection at Ling six sound testing. Furthermore, the TEOAE were bilaterally present, while auditory brainstem responses (ABR) were absent, even at the higher level of stimulation. Tympanometry was bilaterally normal and stapedial reflexes bilaterally absent even at the higher stimulation levels. The audiologic picture was that of an ANSD.

**Figure 1:**
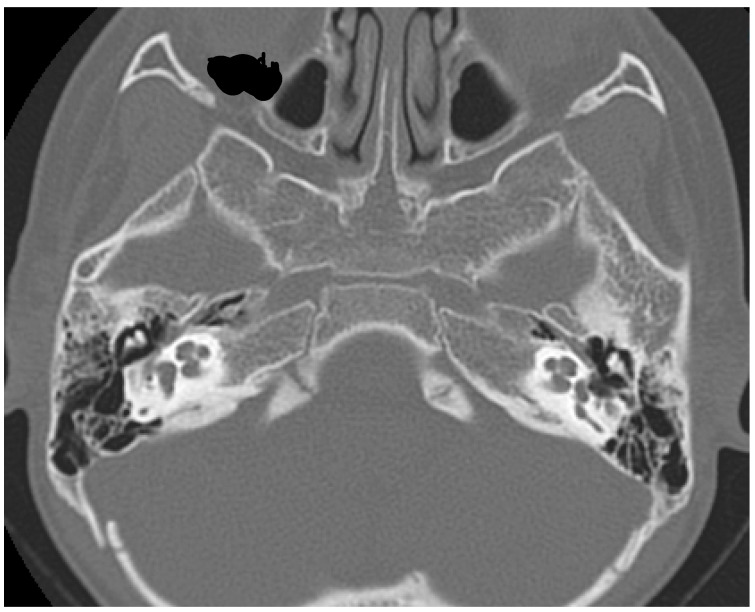
Petrous bone CT showing normal ear anatomy in axial view.

The child was promptly fitted with high power hearing aids bilaterally. After 4 months of traditional hearing aid fitting and speech therapy, with very limited benefit in terms of sounds and speech perception and language development, a CI was proposed. A CT scan of the petrous bone showed normal middle and inner ear anatomy (see Figure 1) and a further MRI confirmed normal anatomy of the labyrinth and cochlear nerves.

**Figure 2:**
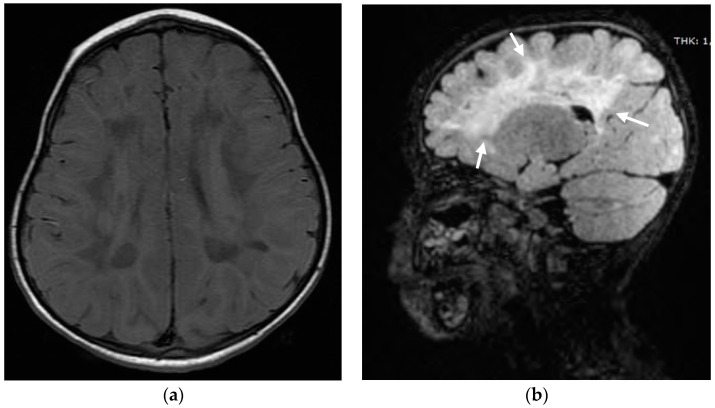
(**a**) Axial T1 weighted sequence. (**b**) Sagittal FLAIR cube weighted sequence, showing leukodystrophy (see white arrows).

At 29 months she presented motor regression with loss of walking, which she had acquired around the age of 25 months. She did not have any infectious and traumatic episodes. At the neurological examinations she manifested fluctuating tone with rigidity on tibio-tarsal joint and hyperactive patellar and Achille’s reflex. A brain magnetic resonance was repeated, which evidenced a worsening of the existing lesions (Figure 2). The developmental neuropsychiatric evaluation successively showed a slow progressive improvement in motor skills. Due to the worsening of the neurological involvement and the possible need to further repeat an MR, the CI procedure was delayed and a unilateral CI on the right ear was performed at the age of 40 months.

**Figure 3:**
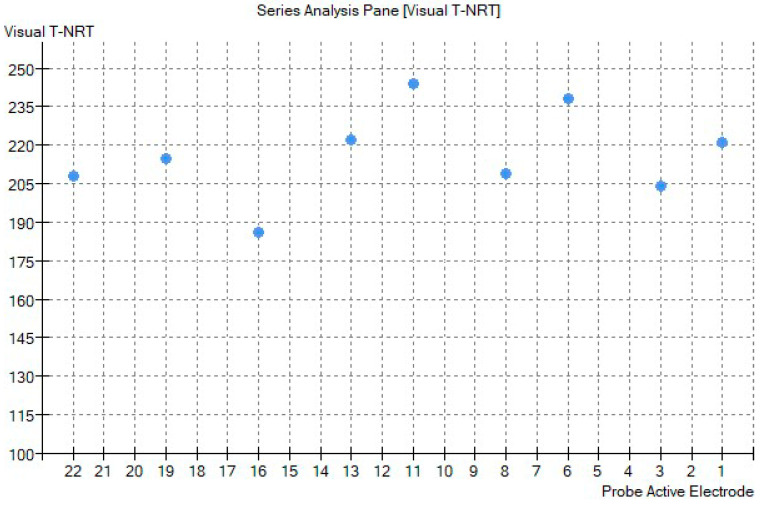
Visual threshold-neural response telemetry (T-NRT) report from intraoperative testing. On the y-axis the amount of Current Unit (CU) is reported.

A Nucleus^®^ CI612 device with a perimodiolar electrode was used. The surgery was uneventful through a standard facial recess approach to the cochlea [12]. Further, intraoperatory telemetry testing evidenced normal impedances on all the electrodes and the neural telemetry testing showed an evocable compound action potential on basal, middle and apical electrodes (Figure 3).

One month after surgery the child underwent the first CI fitting session and continued to attend psychomotor and speech rehabilitation. The speech therapy sessions were only for 1 h a week, due to difficulties the parents had in reaching the hospital. She also underwent periodic CI fitting sessions and audiological follow-up at our institute.

One year after implantation, the child uses the implant for about 7 h per day (as extracted from manufacturer datalogging). Free field threshold with CI is about 30 dB (pure tone audiometry between 0.5 and 1-2-4KHz). The child is able to detect voice, every Ling test phoneme and environmental sounds, and she produces various vocalizations and a few onomatopoeias.

## 3. Discussion

We reported a case of ANSD in PRLTS3, due to CLPP homozygous mutation, diagnosed in a 24-month-old girl, who was submitted to CI at the age of 40 months.

Mutations in CLPP are related with PRLTS3, which presents with progressive hearing loss, female infertility, microcephaly, epilepsy, and growth and mental retardation [2,13,14,15]. Furthermore, it is associated with specific brain MRI anomalies [16].

CLPP is a nuclear encoded mitochondrial protease directed at the mitochondrial matrix. It is encoded on chromosome 19 [17]. This protease participates in mitochondrial protein quality control by degrading misfolded or damaged proteins, thus maintaining the normal metabolic function of the cell. In PRLTS3, the peptidase activity of CLPP is suppressed. The neurological impairments involved in PRLTS3 suggest that the pathogenic mutations in CLPP might trigger a cascade mitochondrial dysfunction [15,17].

In patients with PRLTS, the SNHL is generally bilateral, symmetric, early onset and progressive, very often resulting in a severe-to-profound deficit [5,8,18,19,20]. Some authors have reported cases with an early onset acquired hearing loss that passes the newborn hearing screening [19]. In many cases, the audiometric curves are described to be upsloping [5,8,21] or flat [8,18,19,21,22].

Generally, the hearing loss associated to PRLSTS is reportedly due to a cochlear damage, and very few cases of ANSD have been previously reported. In one case, it was related with biallelic mutation of TWNK gene [7] and in another case with mutation of HARS2 gene [8]. The pathogenic role of heterozygous TWNK mutations have recently been identified in patients with PRLTS5, resulting in hearing loss, ataxia, myopathy, neuropathy and ophthalmoplegia; the recent paper by Oldak and colleagues was the first to reveal a complex background of the SNHL associated with PRLTS5 in two patients, with a partial alteration of cochlear function associated with an auditory neuropathy. Indeed, biallelic variants in HARS2 have been associated with PRTLS2; only in one patient, reported by Demain and colleagues, has the SNHL associated with PRTLS been related to ANSD.

As far as we know, to date, the CLPP mutation detected in the patient herein reported has never been related to ANSD.

It should be observed that, even if ANSD has been found in just two papers [7,8], most of the studies lack a complete audiologic evaluation, including ABR and TEOAE or electrocochleography. Indeed, only four authors reported the cochlear microphonic and/or TEOAE and/or ABR results. Pan and colleagues [5] described a case in which both cochlear microphonics and distortion product otoacoustic emissions (DPOAE) were absent, indicating a cochlear SNHL. Carminho-Rodriguez and colleagues [22] also reported another case of cochlear SNHL with absent transient evoked otoacoustic emission (TEOAE) and present ABR track. Demain et al. [8] and Oldak et al. [7], on the other hand, reported two patients with ANSD, showing the presence of TEOAE associated with an absent ABR track.

It is important to point out that a few other articles also describe unproportionally poor speech discrimination in relation to the hearing deficit degree, in the reported cases [7,23], perhaps underlining the presence of a neuronal involvement.

As far as we know, no cases of ANSD in PRLTS submitted to CI have been reported in literature. In the literature, only six patients with PRLTS and severe-to-profound hearing loss submitted to CI have been reported [8,14,19,21,22], but none of them presented an ANSD; furthermore, no details on post-implantation results are shown and discussed.

For what specifically concerns PRLTS3, in the previous scientific literature 20 cases have been reported. Analysing the audiologic assessment of those cases, in 12 cases the grade of SNHL was profound [2,16], in four cases it was severe-to-profound [14,20,24], in two cases it was reported as severe [21] and in two cases the grade of hearing loss was not reported [13]. The reported diagnosis of hearing loss in PRLTS3 was at birth in eight cases [2,16], and before 3 years of age in four cases [16,20,21]; in one case it was reported that SNHL was diagnosed at 6 years of age [21], while in seven cases the time the hearing loss was diagnosed was not reported [13,14,16]. The result of newborn hearing screening was not reported in any of the cases. A progression of the hearing loss has been reported in four cases [20,21,24]. In none of the cases has the presence of cochlear microphonic or otoacoustic emissions been reported, meaning the possible presence of ANSD. In only one case has the occurrence of a CI been reported, but the results of the procedure were not discussed [14]. The audiologic data of PRLTS3 reported in the literature are summarised in Table 1.

In the case herein reported, a comprehensive audiological assessment was completed at the age of 24 months, defining a picture of ANSD; furthermore, the hearing loss had a delayed onset, since at birth the newborn hearing screening was bilaterally pass both for TEOAE and AABR [25]. The aided and unaided thresholds assessed through behavioural audiometry showed a bilateral severe-to-profound hearing loss and sounds and speech perception with hearing aids was very poor. No language development was appreciable after four months of hearing aid use and speech therapy. This young patient was a candidate for CI procedure, but implantation was delayed until the age of 40 months, due to the worsening of the neurological clinical picture and the need to repeat an MRI.

Some issues related to CI concerning the present case have to be addressed. The first is how a CI can manage to restore hearing in the ANSD and what the intended outcome is. Previous studies showed that individuals with ANSD in which the cochlear sensory system and the synapse are affected, which are bypassed by the CI, have optimal post-implantation outcomes. Besides, also some patients with ANSD due to neural damage benefit from implantation because the stimulus provided by the implant is synchronous and regular, but others show poor performance with CI because of the poor neural transmission of the electrical signal [26,27]. ANSD can also be assessed by electrocochleography showing an absent or abnormal compound action potential, even at high stimulations, and the presence of a robust cochlear microphonic. A detailed diagnosis of the site-of-lesion is also fundamental for the prediction of CI outcomes, which are generally worse in neuropathic patients [28].

Furthermore, in the present case, the results after implantation may be affected both by the neuromotor delay and by the delay in intervention, to the extent that it was only partially pre-operatively predictable [29,30].

The second concern regards the need to submit the patient to serial brain MRI study in the future for the evaluation of the neuroradiologic picture. It is known that, even if the last generation of CI equipped with diametrically bipolar magnets allow pain-free MRI scans even at 3 T and without a headband [31], the magnet itself creates a spherical artifact that makes it hard to see temporal and parietal encephalic structures [32]. In order to limit the artifact and consequently allow a better encephalic evaluation with MRI scans, together with the uncertainty of proper cochlear nerve stimulation by the implant, we decided to proceed with a unilateral right CI. The option of a second sequential implant will be evaluated on the basis of the results of the first implant and of the neurological picture and need of follow-up with MRI.

The choice of the brand of the implant was due to the possibility to modify many stimulation parameters during intraoperative and post-operative neural response telemetry testing and to the possibility to manually adjust the pulse width and other stimulation parameters during the fitting of the implant, which can be useful in the case of a poor nerve.

In the reported case, we demonstrated good neural stimulation by the implant: we recorded stable and consistent compound action potentials with neural response telemetry test on apical, medial and basal electrodes, both intraoperatively and post-operatively during the fitting sessions. We conducted a standard CI fitting procedure, and it was not necessary to use high stimulation parameters, such as high stimulation levels or larger pulse-width.

Since the implant was activated, the patient has been undergoing speech and language and neurodevelopmental disorder rehabilitative treatment once a week for about 12 months. The datalogging of the device showed a constant use of the processor. The sound and speech perception increased significatively, but despite perceptive benefits, to date, speech abilities lag beyond those expected. However, it must be considered that both the delay in intervention and the relevant neurological involvement of the child undoubtfully play a role in limiting the results, even if an effective audiological input is provided.

## 4. Conclusions

To our knowledge this is the first case of ANSD related to PRLTS and CLPP mutation reported in the literature and the first report of the results after CI procedure. Our report adds evidence that CI may be a viable option for cases with ANSD that show unsatisfactory results with traditional hearing aids and particularly in cases affected by PRLTS5.

The need for a comprehensive audiological assessment in paediatric cases with hearing loss is highlighted to completely define the clinical picture, especially in the case of a genetic and syndromic disease; this is important for the global management of the child and also for the audiological and rehabilitative management. Rehabilitative options, including the possibility of a CI, must be evaluated with great attention and on a case-by-case basis, with regard to pre-operative evaluation issues, to issues concerning the choice of the right device, the device fitting and the post-operative rehabilitation and follow-up. In the case herein reported, the presence of a syndromic disease with neuropsychiatric disabilities adds critical aspects to deal with in every phase of the procedure with a multidisciplinary approach.

## Figures and Tables

**Table 1:** Audiologic data of PRLTS3 reported in the literature. SNHL = sensorineural hearing loss; OEs = otoacoustic emissions; ABR = auditory brainstem responses; ANSD = auditory neuropathy spectrum disorder.

Article	Number of Cases	Grade of SNHL	Diagnosis of SNHL	Progression	Result of Newborn Hearing Screening	OEs or Cochlear Microphonic	ABR Track	ANSD	Hearing Restoration
Present article	#1	Severe-to-profound	24 months of age	Yes	Pass	Present	Absent	Yes	Cochlear Implant
Faridi 2021 [24]	#1	Severe-to-profound	Childhood	Yes	Not reported	Not reported	Not reported	-	Not reported
Demain 2017 [20]	#1	Severe-to-profound	14 months of age	Yes	Not reported	Not reported	Not reported	-	Not reported
Dursun 2016 [13]	#2	Not reported (2)	Not reported (2)	Not reported (2)	Not reported (2)	Not reported (2)	Not reported (2)	-	Not reported (2)
Lerat 2016 [21]	#2	Severe (2)	3 years of age (1)	Yes (2)	Not reported (2)	Not reported (2)	Not reported (2)	-	Not reported (2)
			6 years of age (1)						
Theunissen 2016 [16]	#5	Profound (5)	Congenital (1)	Not reported (5)	Not reported (5)	Not reported (5)	Not reported (5)	-	Not reported (5)
			16 months of age (1)						
			Not reported (3)						
Ahmed 2015 [14]	#2	Severe-to-profound (2)	Not reported (2)	Not reported (2)	Not reported (2)	Not reported (2)	Not reported (2)	-	Cochlear implant (1)
									Not reported (1)
Jenkinson 2013 [2]	#7	Profound (7)	Congenital (7)	Not reported (7)	Not reported (7)	Not reported (7)	Not reported (7)	-	Not reported (7)

## Data Availability

All the reported data are available upon reasonable request.
